# Analyzing QCM Data Using a New Transfer-Matrix Model: Long-Ranged Asymmetric Gradient in Shear Modulus Identified Across Immiscible Glassy–Rubbery Polymer Interface

**DOI:** 10.1021/acs.macromol.4c02847

**Published:** 2025-03-19

**Authors:** Alexander A. Couturier, Justin C. Burton, Connie B. Roth

**Affiliations:** Department of Physics, Emory University, Atlanta, Georgia 30322, United States

## Abstract

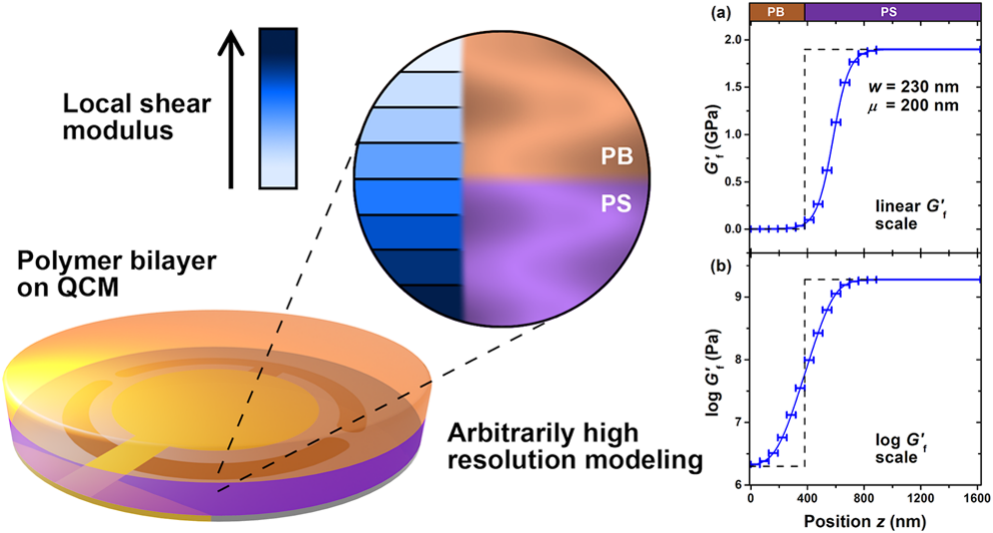

A new approach to analyzing quartz crystal microbalance (QCM) data using an acoustic transfer-matrix model is presented that enables determining a local depth-dependent shear modulus *G̃*(*z*) profile. A strong decrease in dissipation upon annealing is observed for immiscible polymer bilayer films of rubbery polybutadiene (PB) atop glassy polystyrene (PS), reflecting large viscoelastic changes in the sample corresponding to the emergence of a broad gradient in modulus *G̃*(*z*) when the ≈5 nm compositional interface is formed. Using a new transfer-matrix form of our continuum mechanics model that matches boundary conditions of shear waves between discrete modeled layers, we computationally fit these changes in frequency Δ*f*(*n*) and dissipation ΔΓ(*n*) shifts over a range of harmonics *n* to the evolution of a modulus gradient. The *G̃*(*z*) gradient across the PS/PB bilayer, treated as a hyperbolic tangent, is observed to be broad (230 nm) and strongly asymmetric (200 nm) toward the glassy PS side, consistent with the general trends of local glass transition *T*_g_(*z*) previously reported. Surprisingly, the *G̃*(*z*) gradient is found to be symmetric on a log *G* scale, with the value of *G* at the interface equivalent to the geometric mean that optimizes acoustic energy transmission.

## 1 Introduction

Numerous advanced polymer materials today are engineered with multiple polymer components whose morphology have nanoscale structure.^1−8^ The properties of these materials will be heavily dominated by the influence of the interface between the polymer domains. Recent works have demonstrated large and long-range changes in local glass transition temperature *T*_g_ near polymer–polymer interfaces^9−12^ suggesting that the local properties of polymers near interfaces may be far from its anticipated bulk response. An important open question associated with these observations is the extent to which other material properties typically correlated with *T*_g_ are also locally different. In the present work, we specifically aim to address whether the local modulus also changes near glassy–rubbery polymer interfaces in accordance with local *T*_g_ changes. Current efforts in the existing literature measuring modulus changes in thin films are typically limited to measuring an average modulus^13−25^ or interrogating an accessible free or exposed surface.^26−32^ An analysis of existing measurements by Vogt has argued that changes in average modulus with decreasing film thickness *h* are not consistent with a simple shift in the modulus master curve *G̃*(*t, T*) as would be expected from a shift in the average *T*_g_(*h*) of the film, but instead the material appears to behave as a composite reflecting a gradient in modulus with depth from the interface.^17^ Here we present a new transfer-matrix analysis of quartz crystal microbalance (QCM) data to a continuum mechanics model of shear wave propagation through a polymer bilayer film that allows us to determine a depth-dependent profile in shear modulus *G̃*(*z*) across a glassy–rubbery polymer bilayer film.

**Figure 1:**
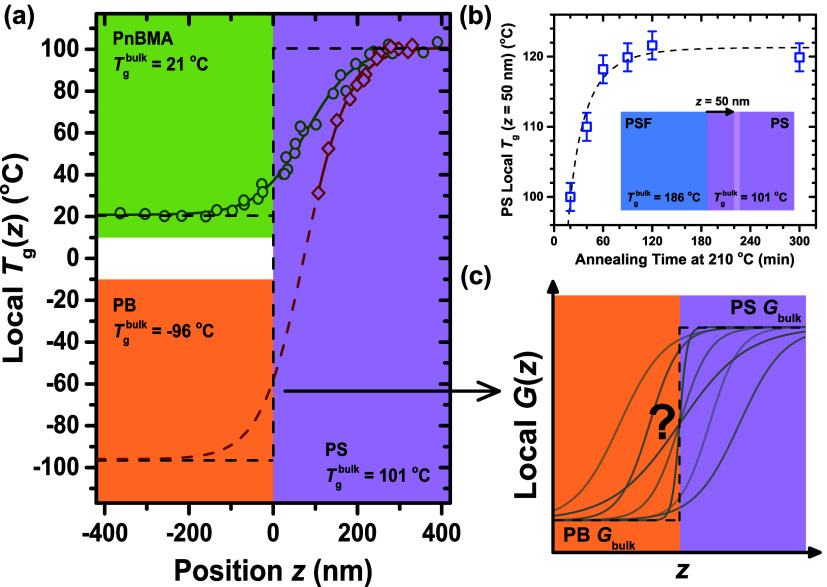
(a) Pyrene fluorescence data from refs (10,12) measuring the local glass transition temperature *T*_g_(*z*) across bilayer films of PS/PnBMA (green circles) and PS/PB (brown diamonds) with equilibrium compositional interfacial widths of ≈5 nm. Curves through the data are best fit tanh profiles showing broad and asymmetric *T*_g_(*z*) gradients spanning ≈200 nm. (b) Local *T*_g_(*z*) data from ref (11) measured in the PS domain at *z* = 50 nm from the interface with PSF as the PS/PSF interface is annealed to its equilibrium compositional interfacial width of ≈6 nm. This demonstrates that the broad *T*_g_(*z*) profile is only established when the glassy–rubbery polymer interface broadens to its equilibrium value. (c) Cartoon graph highlighting the primary question we aim to address with our new QCM analysis technique applied to PS/PB bilayers: what long-ranged gradient in shear modulus *G*(*z*) might exist, and does it correspond to the previously observed long-ranged *T*_g_(*z*) gradient?

Previous works from our lab using pyrene fluorescence have demonstrated long-range gradients in local glass transition temperature *T*_g_(*z*) exist as a function of position *z* across glassy–rubbery polymer interfaces that are, compared to the composition profile, far broader spanning hundreds of nanometers and asymmetric toward the glassy domain.^9−12^ The first set of *T*_g_(*z*) data collected across an interface between poly(*n*-butyl methacrylate) (PnBMA) and polystyrene (PS) is shown in Figure 1a.^10^ The data were found to be well fit by a hyperbolic tangent of the form
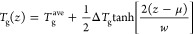
1with a width *w* = 231 ± 5 nm and asymmetry μ = 79 ± 3 nm, compared to the compositional interfacial width *w*_*I*_ = 7 nm.^10^ The average *T*_g_^ave^ = 1/2(*T*_gPS_^bulk^ + *T*_gPnBMA_^bulk^) = 60.8 °C and difference Δ*T*_g_ = *T*_gPS_^bulk^ – *T*_gPnBMA_^bulk^ = 79.9 K in bulk *T*_g_ values where determined from the asymptotic limits of the individual polymers. Similar behavior has been measured for a range of other PS–polymer pairs,^9,11,12,33^ including PS next to polybutadiene (PB) which is also shown in Figure 1a.^12^ A hyperbolic tangent fit to the available PS/PB *T*_g_(*z*) data finds an equally broad and asymmetric profile with *w* = 209 nm and asymmetry μ = 75 nm, assuming *T*_g_^ave^ = 2.5 °C and Δ*T*_g_ = 197 °C calculated from our measured *T*_g_^bulk^ = 101 °C for PS and the manufacturer’s quoted *T*_g_^bulk^ = −96 °C for PB.^12^

A big open question that has been driving the field is to understand the fundamental cause of such long-range gradients.^9,34−41^ Most theoretical efforts have focused on modeling the dynamical gradient near a free surface or substrate interface,^35,42−45^ and when such existing models are adapted to treat soft interfaces the gradient is still predicted to be short-range, ∼10 nm typically decaying exponentially with distance from the interface.^36,38,46,47^ This suggests some additional long-range mechanism that propagates dynamical mobility is needed to explain the long-range *T*_g_(*z*) observations near and across polymer–polymer interfaces.^34,35^ The experimental results themselves have provided insight into what factors are important.^9,34,40,41^ Measurements on different polymer pairs have demonstrated that the compositional interfacial width *w*_*I*_ and modulus of the neighboring domain are key parameters that control the observed *T*_g_(*z*) profile.^9,11,33^ Local *T*_g_(*z*) data collected in PS at a distance of *z* = 50 nm from an interface with polysulfone (PSF) (*T*_g_^bulk^ = 186 °C) at progressively increasing annealing times at 210 °C demonstrated how the perturbation to local *T*_g_(*z* = 50 nm) occurred and saturated as the polymer–polymer interface evolved to equilibrium (*w*_*I*_ = 6 nm for PS/PSF), as illustrated in Figure 1b.^11^ In contrast, *T*_g_(*z*) measurements in PS next to polydimethylsiloxane (PDMS), a highly immiscible polymer pair that has a much smaller equilibrium compositional interfacial width (*w*_*I*_ ≈ 1.5 nm), exhibited a significantly shorter *T*_g_(*z*) perturbation distance of *z* = 65–90 nm before bulk *T*_g_ was recovered.^33^ Across different measurements, we find that a breadth in the compositional interface of ≈5 nm appears to be necessary to strongly couple the dynamics between the two different polymer domains.^9,34^ Most studies that investigate the modulus of glassy PS next to a rubbery layer like PDMS typically do not or cannot anneal the polymer–polymer interface together.^13,18−20,23,24^ As a result the interface will remain sharp (≲1 nm), which we believe is likely the reason why minimal perturbation from the glassy–rubbery polymer interface is observed in such studies.^9^ Our investigation of the PS/PDMS system also demonstrated that *T*_g_(*z* = 50 nm) could be shifted by 40 K simply by adjusting the cross-link density of the PDMS to vary its modulus between *E* = 0.9–2.6 MPa.^33^ This indicates that the difference in modulus between the two polymer domains is also a strong factor altering the degree to which the dynamics are coupled between them.

Most recently, Gagnon et al. has leveraged the QCM as a MHz-frequency rheometer to demonstrate that a large change in viscoelastic properties occurs across a glassy–rubbery PS/PB bilayer film as the PS/PB polymer interface is formed.^34^ The QCM resonances at the higher harmonics that are sensitive to the viscoelastic properties of the film were shown to exhibit a large reduction in dissipation upon annealing at elevated temperature as the PS/PB interface formed, evolving from *w*_*I*_ ≈ 1 nm to ≈ 5 nm. Analysis was done with a physically intuitive continuum mechanics model describing the QCM shear wave propagation across the bilayer sample that was previously shown to determine frequency dependent storage *G*′(*f*) and loss *G*″(*f*) moduli in agreement with time–temperature shifted rheometry data.^48^ The study showed that a simple two-layer model worked well to describe the initially assembled bilayer film with *w*_*I*_ ≈ 1 nm, but failed to reasonably describe the PS/PB bilayer response on annealing as *w*_*I*_ grew to ≈5 nm.^34^ A trilayer model was implemented to fit the QCM data that assumed an intermediate layer between the PS and PB domains with average modulus , leaving a single fitting parameter corresponding to the thickness of this intermediate layer that grew up to ≈180 nm on annealing at 120 °C for 100 min. These results suggest that a broad gradient in modulus develops between the glassy PS and rubbery PB domains as the glassy–rubbery polymer interface is formed.^34^ However, the simplistic trilayer model used was unable to comment on whether any asymmetry was present in this effective modulus gradient, as is observed in the *T*_g_(*z*) gradients. The paper by Gagnon et al. did propose a new idea of how local material properties could be coupled over large distances across different polymer domains with broad compositional interfacial widths (*w*_*I*_ ≈ 5 nm).^34^ Via acoustic impedance matching, high frequency acoustic waves ∼300 GHz and greater (λ ≲ 5 nm) would be expected to transmit across such a broad compositional interface, coupling the vibrational density of states (VDoS) at frequencies around the boson peak between the two domains.^34^ Such high frequency acoustic waves could then interact with localized soft collective excitations potentially triggering density fluctuations and associated α-relaxation events over long distances.^38,49−51^

The present work aims to better understand the gradient in modulus *G̃*(*z*) that emerges upon annealing of the glassy–rubbery PS/PB polymer interface and assess the degree to which it tracks with the previously measured asymmetric gradient in *T*_g_(*z*). We leverage a transfer-matrix approach to expand the analysis of our continuum mechanics model for QCM data to an arbitrary number of modeled layers, allowing for an essentially continuous *G̃*(*z*) gradient to be identified. We show that the large change in dissipation exhibited by the films upon annealing for 40 min at 120 °C is best fit by a *G̃*(*z*) gradient that is 230 nm wide, despite the compositional interface spanning only ≈5 nm. To fit the QCM data well, we find the gradient must also be highly offset toward the PS side, with a best fit asymmetry term of 200 nm, corroborating the strong asymmetry toward the PS side found in previous local *T*_g_(*z*) measurements in this system.^9,12^ This asymmetry corresponds to a local modulus value at the PS/PB interface far below the average of the two bulk modulus values. However, our best fit *G̃*(*z*) profile places the local modulus value at the interface extremely close to the geometric mean of the bulk values, corresponding to a *G̃*(*z*) gradient that is symmetric on a logarithmic modulus scale. The geometric mean happens to be the optimal condition for impedance matching of acoustic waves, which may have profound implications for our understanding of the mechanisms of local properties coupling across interfaces.

## 2 Experimental Methods

Polystyrene (PS) (*M*_w_ = 403 kg/mol, *M*_w_/*M*_n_ = 1.02) and polybutadiene (PB) (*M*_w_ = 375 kg/mol, *M*_w_/*M*_n_ = 2.4; 36% cis 1,4; 55% trans 1,4; 9% vinyl 1,2, as specified by the supplier) were purchased from Scientific Polymer Products. The PS was used as received, while the PB was washed three times by dissolving in tetrahydrofuran and precipitating in chilled methanol in order to remove any plasticizer present, and then subsequently stored in a refrigerator.^12^ Films were constructed by spin-coating^52^ from toluene solutions onto freshly cleaved mica. Films were then each cut into two parts, one to be floated onto a silicon substrate for a film thickness measurement by ellipsometry,^53^ and one to be floated onto a QCM sensor.

Films were floated onto the QCM sensor such that there was full coverage, and any part of the film that got onto the backside was carefully and thoroughly wiped off with toluene. The thicknesses of the PS layers were *h*_PS_ = 1230 ± 20 nm and the PB overlayers were *h*_PB_ = 360 ± 30 nm. These layer thicknesses were chosen for the QCM measurements based on a balance between being thick enough to obtain sufficient modulus sensitivity, while being thin enough that there is not so much dissipation that an insufficient number of harmonics is measurable. The PS underlayers were annealed onto the QCM at 120 °C (above *T*_g_^PS^) under vacuum for approximately 20 h to remove any residual solvent and release stresses developed during spin-coating. The PB overlayers were held under vacuum on the PS+QCM system at room temperature (above *T*_g_^PB^) for ≈20 h for the same reasons. Importantly, this 20 h period of time at room temperature under vacuum allows the PS/PB interface to establish a minimum baseline width that should be at most ≈1 nm.^54,55^ We define this sample state as the *t* = 0 initial condition prior to annealing at 120 °C. QCM measurements were performed on these samples at the initial *t* = 0 state, as well as after annealing the PS/PB bilayer for different durations at 120 °C under vacuum.

Ellipsometry measurements were performed using a J. A. Woollam M-2000 variable angle spectroscopic ellipsometer with a rotating compensator. The film thickness is obtained by fitting the complex ratio of reflected *p*- to *s*-polarized light to an optical layer model. The model treats the polymer film as a transparent Cauchy layer  atop a semi-infinite silicon substrate with a 1.25 nm native oxide layer.^53^

The QCM measurements were performed using AT-cut quartz sensors purchased from Inficon that each have a fundamental frequency *f*_f_ of 5 MHz. This is the resonance frequency of the bare QCM at the first harmonic, the exact value of which is measured before the construction of every sample. Resonance peaks were collected with a vector network analyzer (Agilent 4395a) driving the system at 0 dBm, corresponding to a power of 1 mW. The measurements were all performed at room temperature (25 °C). For each harmonic *n*, the peak position in the resonance trace is the resonance frequency *f*_*n*_ and the bandwidth of the peak is proportional to the dissipation Γ_*n*_. The amplitude of the resonance peak generally decreases as bandwidth increases. Extracting exact values of *f*_*n*_ and Γ_*n*_ using circuit relations follows the protocol of our previous work on QCM measurements,^34,48^ but now using a Python library (lmfit) to perform the fitting.^56^ The experimental data are the shifts in the resonance resulting from adding the polymer film: Δ*f*_*n*_ = *f*_*n*_^film+QCM^ – *f*_*n*_^bare QCM^ and ΔΓ_*n*_ = Γ_*n*_^film+QCM^ – Γ_*n*_^bare QCM^. It should be noted that Δ*f*_*n*_ and ΔΓ_*n*_ are always collected over a range of harmonics, typically *n* = 1, 3, 5, 7. To keep the notation compact, the explicit *n* subscript is dropped for Δ*f* and ΔΓ in the subsequent discussion unless a specific resonance is being referred to.

**Figure 2:**
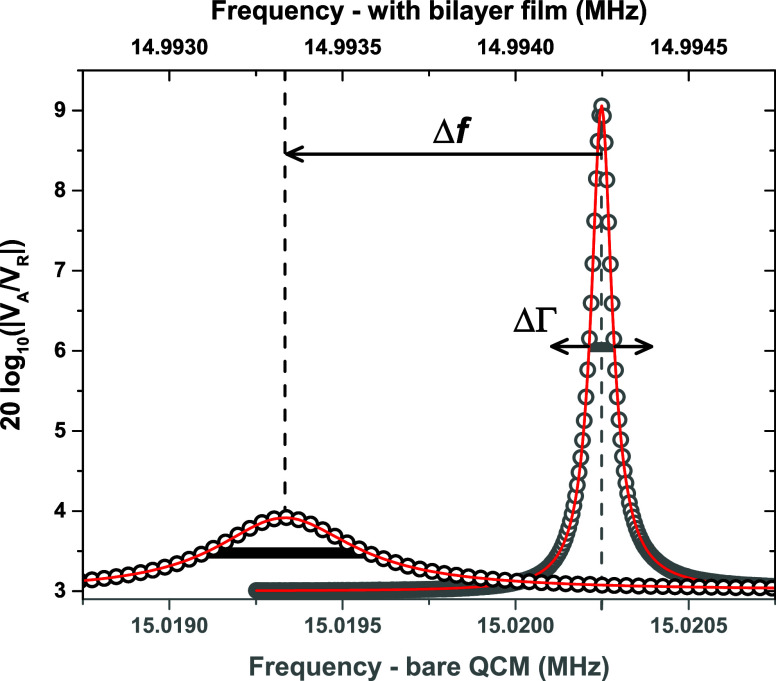
Measured resonance traces at *n* = 3 (15 MHz) are plotted for a bare QCM (gray data along the bottom horizontal axis) and for the subsequent addition of the PS/PB bilayer film onto the QCM (black data along the top horizontal axis). Red curves are fits of the resonance traces to the functional form corresponding to our QCM circuit relations used to determine *f*_*n*_ and Γ_*n*_.^48^ Both horizontal axes have a range of 2 kHz to keep relative bandwidths to scale. The black trace’s data points are less vertically spread out, so half of the data points are hidden in the black trace to visually distinguish them.

Figure 2 provides an example of a resonance trace at *n* = 3, before and after assembling a PS/PB bilayer film atop the QCM. The resonance traces are measured by the network analyzer connected to the QCM, reading the voltages *V*_A_ and *V*_R_. The ratio of these voltages produces the amplitude on the vertical axis. Following circuit relations previously established,^48^ these resonance traces are fit to extract Δ*f* and ΔΓ, the distilled resonance data of interest. The data shown in Figure 2 are for a 352 nm PB layer atop a 1222 nm PS layer at the *t* = 0 sample condition. The initial resonance frequency *f*_3_^bare QCM^ and dissipation Γ_3_^bare QCM^ of 15.020250 MHz and 26 Hz for the bare quartz are shifted to *f*_3_^film+QCM^ = 14.993331 MHz and Γ_3_^film+QCM^ = 233 Hz by the addition of the bilayer film, giving Δ*f*_3_ = −26.919 kHz and ΔΓ_3_ = 207 Hz. Similar data are collected for the other harmonics *n* = 1, 5, 7 giving the set of Δ*f*(*n*) and ΔΓ(*n*) data for this sample state, where the procedure is then repeated at different time points after annealing the PS/PB bilayer at 120 °C.

## 3 Results and Discussion

The goal of this work is to use a new transfer-matrix analysis of QCM data to determine what gradient in modulus *G̃*(*z*) emerges during the formation of an immiscible glassy–rubbery polymer interface. We focus on a bilayer system of high molecular weight polybutadiene (PB) and polystyrene (PS) for which previous work has demonstrated that a broad and asymmetric gradient in local *T*_g_(*z*) occurs across the PB/PS interface when fully annealed to its ≈5 nm equilibrium value.^54,57^ To maximize comparison to previous experimental work, while optimizing the viscoelastic sensitivity of the QCM, the sample geometry selected for study was a glassy PS layer of thickness *h*_PS_ ≈ 1250 nm capped with a rubbery PB overlayer of *h*_PB_ ≈ 350 nm. From our QCM modeling, we determined that a minimum of ≈1000 nm would be needed for the PS layer to ensure enough precision to measure its glassy modulus prior to adding the PB layer, while the rubbery PB layer could reasonably be as thick as 500–600 nm before dissipation is too large to adequately resolve the higher harmonics.^48,58,59^

Specifically, we are interested in experimentally characterizing how the viscoelastic response of the PS/PB bilayer film changes on annealing at 120 °C. We start from the initial *t* = 0 PS/PB bilayer state that has only been held at room temperature for ≈20 h under vacuum. The interfacial width for this glassy–rubbery bilayer system should be limited to ≈1 nm because room temperature is well below the bulk *T*_g_ of the glassy PS, *T*_g_ = 100 °C.^54,55^ The PS/PB bilayer sample is then annealed at 120 °C under vacuum for different lengths of time that grow the interfacial width toward its equilibrium value of ≈5 nm.^54,57^ Based on previous *T*_g_(*z*) measurements by fluorescence, this small increase in interfacial width between the PS/PB domains to its equilibrium value appears to be responsible for establishing the broad *T*_g_(*z*) profile shown in Figure 1a.^11,12^ The question we are aiming to address with the present measurements is, to what extent is there also a gradient in local modulus *G̃*(*z*) established between the PS/PB domains?

In a recent study by our group, a simple three-layer model was used to model the viscoelastic properties of PS/PB bilayers.^34^ This simple analysis suggested the emergence of a broad modulus profile across the PS/PB interface on annealing at 120 °C, but had no ability to model a depth-dependent modulus *G̃*(*z*). In the present work, we introduce an acoustic transfer-matrix model entirely written in Python code that divides the QCM bilayer sample into an arbitrary number of modeled layers. The oscillation of the QCM establishes a standing shear wave across the PS/PB bilayer sample at the given resonance of interest. The behavior of the shear wave within the sample is described by matching the boundary conditions of continuous displacement and stress at the boundary between each modeled layer. From this analysis, we are able to fit the experimental Δ*f* and ΔΓ data collected at the range of harmonics *n* = 1 – 7 in order to determine a depth-dependent modulus gradient across the PS/PB bilayer. The data are fit as a combined data set with Δ*f* and ΔΓ as a function of *n*.

**Figure 3:**
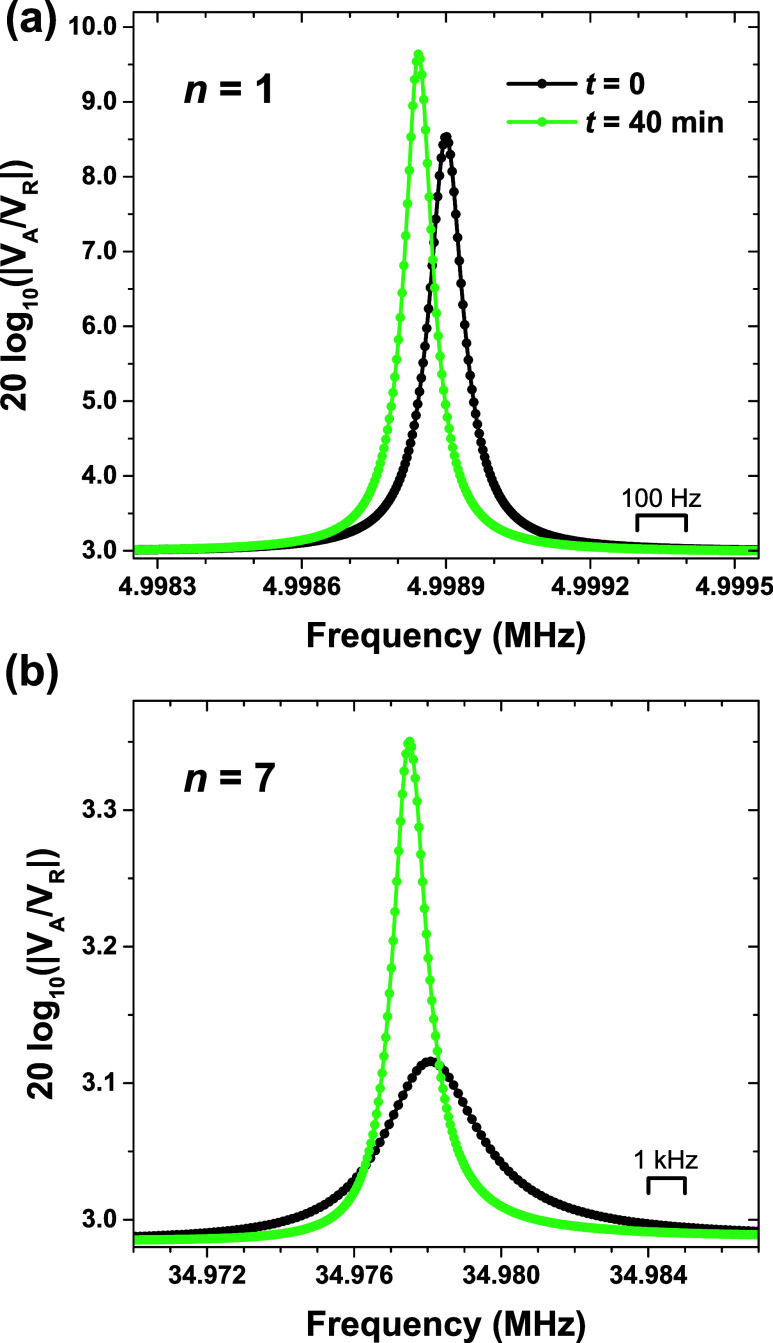
A comparison of resonance traces collected from PS/PB bilayer films at *t* = 0 and *t* = 40 min of annealing at 120 °C. (a) *n* = 1 resonance shows a change from Δ*f* = −8.932 kHz and ΔΓ = 14 Hz at *t* = 0 to Δ*f* = −8.989 kHz and ΔΓ = 7 Hz at *t* = 40 min. (b) *n* = 7 resonance shows a change from Δ*f* = −64.222 kHz and ΔΓ = 1496 Hz at *t* = 0 to Δ*f* = −64.740 kHz and ΔΓ = 475 Hz at *t* = 40 min.

We start by comparing the resonance traces of the PS/PB bilayer measured at the *t* = 0 initial condition with that collected at *t* = 40 min of annealing at 120 °C. Figure 3 shows the resonance traces observed at the *n* = 1 and *n* = 7 harmonics at these two time points for the same representative sample from Figure 2. The annealing at 120 °C results in a small change in bilayer composition where the PS/PB interfacial width grows from ≈1 nm to ≈5 nm, yet Figure 3 demonstrates a large change in the viscoelastic properties of the PS/PB bilayer sample. The changes are substantially greater at *n* = 7, where the QCM is much more sensitive to the viscoelastic response of the film. From these resonance traces we determine the change in Δ*f* and ΔΓ upon annealing at 120 °C, as these values will form the experimental data to be fit by the acoustic transfer-matrix model. The *n* = 7 resonance in Figure 3 shows a large decrease in dissipation upon annealing at 120 °C from ΔΓ = 1496 Hz to ΔΓ = 475 Hz, reflecting a large change in viscoelastic properties of the PS/PB bilayer indicative of the QCM shear waves losing less energy as they pass through the sample. In most of the subsequent discussion, we will continue to focus our analysis on these two time points at *t* = 0 and *t* = 40 min of the PS/PB bilayer system.

### 3.1 New Acoustic Transfer-Matrix Model for QCM Data

We model the QCM-film system using a continuum mechanics approach for the linear viscoelastic response of the polymer bilayer film with a frequency dependent shear modulus *G̃*(ω) = *G*′(ω) + *iG*″(ω). This work builds on our recent physically intuitive continuum mechanics model that predicts the shifts in the resonance frequency Δ*f* and dissipation ΔΓ of the QCM with the addition of the polymer film to determine the polymer’s storage *G*′(ω) and loss *G*″(ω) modulus.^48^ The basis of this model is a description of the QCM shear wave behavior across the sample, fulfilling the boundary conditions of continuity of displacement (no slip) and continuity of stress at each interface. Similar to most QCM modeling approaches, the QCM resonator is treated as an infinite parallel plate where oscillations along only one of the axes of the plane need to be considered.^59^ Our previous work on single layer polymer films showed that this physically intuitive continuum mechanics model can be used to measure *G*′(ω) and *G*″(ω) at MHz frequencies in agreement with time–temperature shifted rheometry data.^48^ In a recent study using a coarse three-layer model, we found evidence that a wide region of intermediate modulus spanning 100+ nm across the glassy–rubbery interface of a PS/PB bilayer film emerges upon annealing.^34^ In the present work, we demonstrate how the physically intuitive continuum mechanics model can be expanded using an acoustic transfer-matrix approach to emulate a depth-dependent shear modulus *G̃*(*z*). This new approach allows us to identify localized depth-dependent information about the system’s viscoelastic properties.

**Figure 4:**
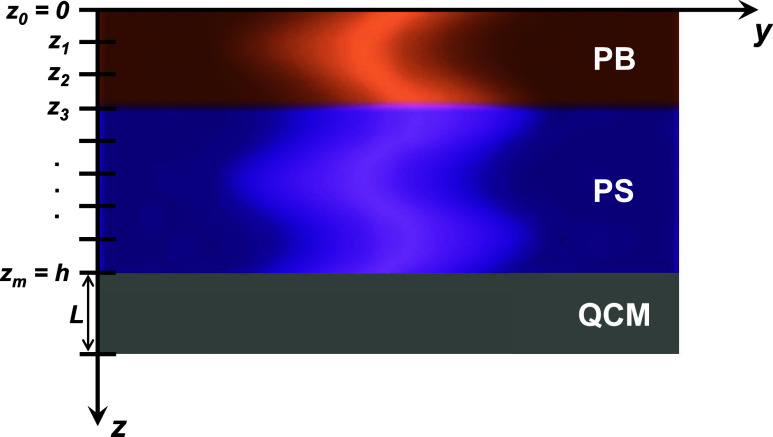
Schematic of the PS/PB bilayer film computationally divided up into *m* discrete layers. Shear waves, oscillating along the *y*-axis, propagate in the *z* direction, where *z* = 0 is defined at the top film/air interface. The total polymer film has thickness *h* = *h*_PB_ + *h*_PS_, and the QCM has a thickness *L*.

The transfer-matrix formalism is most commonly used in optics to describe the propagation of electromagnetic waves through a stratified medium,^60^ although similar mathematical approaches have been previously applied to QCM analysis usually in the context of impedance matching.^61−63^ Here, we apply a transfer-matrix formalism to the propagation of acoustic shear waves across the QCM-film system, where a matrix product relates the wave amplitude coefficients at each interface. By expanding the math into a matrix product series, the polymer bilayer film can be divided up into as many discrete layers as desired to model a depth-dependent shear modulus *G̃*(*z*) with the needed resolution to emulate a continuous gradient. Figure 4 shows how the polymer bilayer film of total thickness *h* = *h*_PB_ + *h*_PS_ is divided up into a series of *m* discrete layers. The horizontal oscillations caused by the QCM are along the *y*-axis, while the shear wave propagation perpendicular to the plane of the QCM is along the *z*-axis. We chose to define *z* = 0 at the top of the polymer bilayer film with the *z*-axis pointing downward into the film because this optimizes computations by imposing full reflection of shear waves at the top air interface. As the air is only able to impart negligible stress to the system, full reflection is expected at the film/air interface (*z* = 0).^48^ The shear wave displacement *u⃗*(*z, t*) in the system is described by the equation
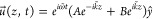
2propagating along the *z*-axis and displacing molecular structure along the *y*-axis. At a given harmonic resonance, a standing shear wave is established from waves propagating in the forward (+*z*) and backward (−*z*) directions, where *A* and *B* are the amplitude coefficients associated with the shear waves at the given frequency propagating forward and backward, respectively. The complex wavenumber  contains the physical properties of the layer, the medium’s density ρ and the complex shear modulus *G̃* = *G*′ + *iG*″. The complex angular frequency
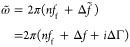
3contains the frequency and dissipation shifts Δ*f̃* = Δ*f* + *i*ΔΓ, caused by the addition of the polymer film, relative to the idealized resonant frequency *nf*_*f*_ of the QCM at the harmonic *n*. To account for variations between different resonators, the fundamental frequency *f*_*f*_ is measured experimentally for each bare QCM crystal prior to adding polymer layers. The relevant component of the stress tensor σ_*yz*_ for the shear wave oscillating in the *y*-direction applying a force to a cross-sectional plane with a surface normal along the *z*-axis is^48^
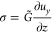
4

The system of equations to be solved is obtained by matching boundary conditions at each interface between the layers in the model. The PS/PB bilayer film is divided up into a total of *m* layers, where we index the layers and their properties with *j* starting from *j* = 0 at the top of the film. We define *z*_*j*_ as the position at the top of layer *j*. The *m*^th^ layer is associated with the QCM, so there are *m* + 1 total layers counting the QCM since *j* starts from zero.

Continuity of stress imposes zero stress at the air interfaces. For the air interface at the top of the film *z*_0_ = 0, the boundary condition applied to the properties of layer *j* = 0 is
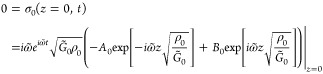
resulting in
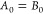
5Equation 5 follows from differentiating eq 2 according to eq 4, evaluated at *z* = 0. At the bottom air interface, the properties of layer *j* = *m* (the QCM) are evaluated at position *z* = *h* + *L*, where *h* + *L* is the thickness of the PS/PB film plus the QCM. Similarly imposing continuity of stress at the bottom air interface, the boundary condition is
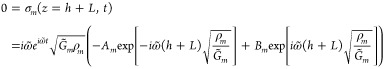
yielding
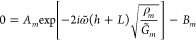
6

The boundary conditions of continuity of displacement and stress at the internal interfaces provide relations of the form
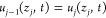
7
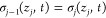
8which correspond to layers *j* and *j* – 1 at position *z*_*j*_. These relations are a system of coupled equations with *A*_*j*_ and *B*_*j*_ coefficients that can be written in matrix notation:
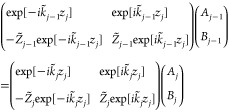
9The top row of the matrix product on each side of eq 9 captures the fact that two adjacent model layers at the same location *z* have the same displacement, while the bottom row captures that they have the same stress at location *z*. To keep eq 9 more compact,  is expressed as impedance *Z̃* and  as wavenumber *k̃*. Note, the time-dependence cancels out similarly to eqs 5 and 6.

Equation 9 corresponds to the multiplication of 2 × 2 matrices connecting layer *j* – 1 to layer *j*, which we can denote simply as *M*_*j*–1_^top^ and *M*_*j*_^bot^, with the top and bot superscripts identifying whether the matrix is associated with the layer on the top side or the bottom side of the model interface being evaluated. Thus, eq 9 can be neatly written as
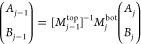
10Iterating through the layers by incrementing *j* from 1 to *m*, eq 10 can be used to connect the parameters *A*_*m*_ and *B*_*m*_ at the film/QCM interface with the parameters *A*_0_ and *B*_0_ at the top of the film, forming the product series
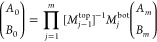
11

The matrix product series of eq 11 is, upon evaluation, a 2 × 2 matrix that can be compactly written yielding
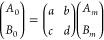
12By matrix multiplication, this gives
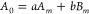
13
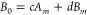
14From the boundary condition eq 5, we have that *A*_0_ = *B*_0_ such that
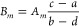
15We then substitute this into the boundary condition eq 6 to ultimately arrive at
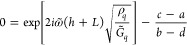
16where *A*_*m*_ nicely cancels out.

Equation 16 becomes the central equation to be solved in relating *G̃* to the resonance shifts Δ*f* and ΔΓ, which are contained in ω̃ according to eq 3. The amplitude coefficients are no longer involved, and instead we have the matrix elements *a*, *b*, *c*, and *d* that each contain long expressions resulting from inverting and multiplying the matrices of the form shown in eq 9. These matrix elements contain the details of how the depth-dependence of *G̃*(*z*) is parametrized to describe the viscoelastic properties of the PS/PB bilayer film.

The modeling process is done by a Python script we built that generates the product series in eq 11 by iterating the matrix operations for a specified arbitrary number of layers *m*. The behavior of the depth-dependent modulus *G̃*(*z*) at each harmonic *n* across these *m* layers is described by a function of *z* with parameters that will be fit by comparing the model-determined Δ*f* and ΔΓ values for a given *G̃*(*z*) profile to the experimental Δ*f*(*n*) and ΔΓ(*n*) data. Newton root-finding is used to obtain Δ*f* and ΔΓ from eq 16, and a Levenberg–Marquardt fitting algorithm^56^ is used to fit the functional form of *G̃*(*z*). We verified that the Newton root-finding algorithm^64^ accurately converges on the single complex root Δ*f̃* = Δ*f* + *i*ΔΓ of eq 16 by mapping out the expression output as a function of Δ*f* and ΔΓ over a wide range of *G̃*(*z*) parameter values.

#### 3.1.1 Modeling Depth-Dependent Modulus Gradient

The frequency dependence of the complex modulus *G̃* is comprised of four separate components that approximate *G*′(ω) and *G*″(ω) as being linear on log–log axes, as the measured QCM frequency range is small (*f* = 5 – 35 MHz for *n* = 1 – 7):^48^
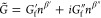
17*G*_f_^′^ and *G*_f_^″^ refer to the storage and loss shear moduli at the first harmonic *n* = 1. β′ and β″ are the slopes of log(*G*′) versus log(*f*) and log(*G*″) versus log(*f*) over the relevant narrow frequency range. This approximation of linear behavior in log(*G*′) and log(*G*″) was previously shown to work well matching QCM results with time–temperature shifted rheometry data for single layer PS and PB films.^48^

To implement a depth-dependent modulus with a broad gradient, we need a functional form for *G̃*(*z*) that can describe *G̃* at each layer in the model with a concise parametrization. Given that previous measurements shown in Figure 1 of the local depth-dependent *T*_g_(*z*) were well described by a hyperbolic tangent, eq 1, a reasonable starting point for *G̃*(*z*) is a similar hyperbolic tangent functional form:
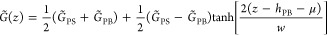
18Note for the QCM analysis, *z* = 0 corresponds to the top of the bilayer sample at the PB free surface. In eq 18, the thickness of the PB layer *h*_PB_ has been subtracted from the position *z* to adjust the coordinate system for the tanh evaluation such that the asymmetry μ is still relative to the PS/PB interface. The limiting values of the hyperbolic tangent are described by the known bulk values of the pure components *G̃*_PS_ and *G̃*_PB_, leaving only two parameters describing the width *w* and asymmetry μ of the gradient. The use of a hyperbolic tangent function is justified over the use of simpler functions such as a line connecting the bulk values because physical systems generally do not have gradients in properties with discontinuous derivatives. As for not using alternative sigmoidal forms such as a Gauss error function, the difference between such functions and an analogous hyperbolic tangent is not sufficiently large that our approach has the sensitivity to distinguish it. Use of the hyperbolic tangent makes comparison to a step change at the compositional interface easy to understand by analyzing the proximity of *w* and μ to zero. It also allows us to address a central question in the field regarding the extent to which local properties like *T*_g_ and *G̃* are correlated. The gradient *G̃*(*z*) can be visualized with plots of *G*′(*z*) or *G*″(*z*) at a given harmonic of choice.

Similar to *G̃*(*z*) the depth-dependent density ρ(*z*) is also modeled as a hyperbolic tangent. However, given that much literature has demonstrated a decoupling between density and dynamics for interfacial perturbations,^35,65−69^ we choose to hold the density profile at the known sharp composition profile of the PS/PB bilayer^54,57^ with density values for the bulk limits of PS and PB taken from the literature.^70−73^ Therefore, the parameters in this model are the PS and PB layer thicknesses *h*_PS_ and *h*_PB_, and the parameters describing the depth-dependent modulus gradient *G̃*(*z*): *G̃*_PS_, *G̃*_PB_, *w*, and μ. In the next section, we describe the set of initial measurements done on each sample that defines *h*_PS_ and *G̃*_PS_ for the PS layer, followed by *h*_PB_ and *G̃*_PB_ for the PB layer, leaving only *w* and μ to evolve with annealing of the PS/PB bilayer at 120 °C.

### 3.2 Analysis of Experimental QCM Data from PS/PB Bilayer Films

#### 3.2.1 Initial Set of Measurements to Define Starting Resonance and Bulk Limits

For each sample, we start by measuring the resonance of the bare crystal, followed by the resonance shift after adding the PS layer. This allows us to determine the PS layer thickness *h*_PS_ and the modulus of the PS layer *G̃*_PS_ using the QCM model with *m* = 1 layer applied to the single layer PS film. Next, the PB layer is added on top, followed by a period where this sample is held at room temperature under vacuum for 20 h to establish an initial well-defined PS/PB bilayer film with a sharp interfacial width of ≈1 nm.^54,74^ This initial bilayer corresponding to our *t* = 0 initial condition is fit to the QCM model with *m* = 2 layers to determine the PB layer thickness *h*_PB_ and the modulus of the PB layer *G̃*_PB_. Thus, from this set of initial measurements for every sample, all parameters except the width *w* and asymmetry μ describing the shape of the modulus profile *G̃*(*z*) are defined. Six nominally identical samples were prepared, where below we report the averaged values across these six samples.

For the PS layer, the modulus *G̃*_PS_ is treated as frequency independent in eq 17 such that β′ and β″ are held fixed at zero, as was done previously based off of literature rheometry data time–temperature shifted to QCM frequencies.^48^ For β′, this is a reasonable assumption based on the very weak frequency dependence of *G*′ for PS when it is very far into its glassy state in the MHz regime.^75,76^ As for β″, *G*″ is difficult to measure with rheometry so far into the glassy state, thus it does not have a clear trend with increasing frequency up from 5 MHz. Regardless, *G*″ is orders of magnitude smaller than *G*′, and hence has a relatively minor impact on what is observed with the QCM. This means that assigning a nonzero value to β″ is not worth the extra fitting parameter, as any impact it has is well within the uncertainty. The PS density ρ is held fixed at 1040 kg/m^3^. This is keeping consistent with what was used previously,^34,48^ which was informed by literature values ranging from 1040 to 1052 kg/m^3^.^70−72^ Fitting *h*_PS_, *G*′, and *G*″ up to resonance *n* = 15 for the PS underlayers, averaged over six samples, resulted in *h*_PS_ = 1240 ± 20 nm, *G*_f_^′^ = 1.9 ± 0.3 GPa, and *G*_f_^″^= 70 ± 40 MPa. *h*_PS_ is in excellent agreement with the ellipsometry measurements showing 1230 ± 20 nm. *G*_f_^′^ and *G*_f_^″^ are consistent with our previous QCM measurements, as well as the ranges indicated in the literature of ≈1.0–1.7 GPa for *G*_f_^′^ and ∼10–50 MPa for *G*_f_^″^.^70,71,75,76^ The high uncertainty in *G*″ for PS is expected given its small value compared to *G*′, and it is consistent with the imprecise range of values indicated by the literature at these frequencies.

Adding the PB layer and establishing the *t* = 0 state, the bulk properties of the PB overlayer are determined from a two-layer (*m* = 2) analysis of this PS/PB bilayer system. The frequency dependence of the PB modulus *G̃*_PB_ following eq 17 has β′ and β″ held fixed at 0.5 and 0.74, respectively, according to the analysis we had previously done on PB master curves from the rheometry literature.^48,73,77−79^ The PB density ρ is held fixed at 895 kg/m^3^, again based on the literature.^73^ This analysis of the PB overlayers using resonances up to *n* = 7 found *h*_PB_ = 380 ± 20 nm, *G*_f_^′^ = 2 ± 2 MPa, and *G*_f_^″^ = 3.3 ± 0.7 MPa, from the average of six samples. The *h*_PB_ value agrees very well with the ellipsometry measurements that gave 360 ± 30 nm. *G*_f_^′^ and *G*_f_^″^ agree with our group’s previous single-layer^48^ and bilayer^34^ QCM measurements, along with additional single-layer PB QCM measurements that we performed, which are all consistent with the time–temperature shifted literature range of *G*_f_^′^ ≈ 2.5–3.5 MPa and *G*_f_^″^ ≈ 2.5–4.5 MPa.^73,77−79^

**Figure 5:**
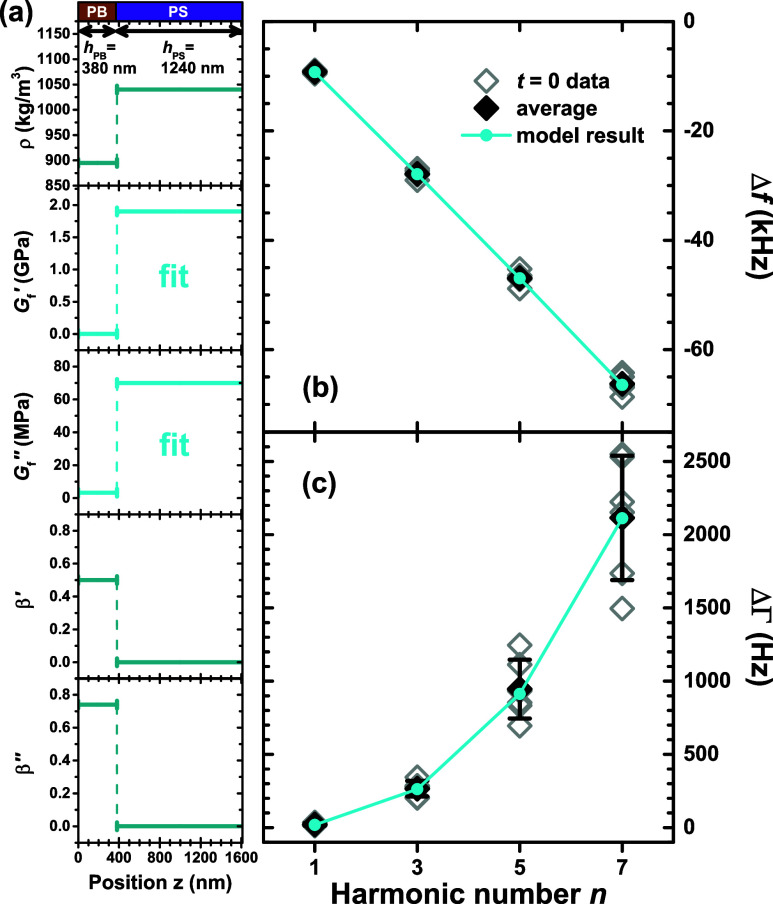
QCM transfer-matrix model applied to the frequency Δ*f*(*n*) and dissipation ΔΓ(*n*) shifts measured for PS/PB bilayer films at *t* = 0 where the interfacial width is sharp (≈1 nm). (a) Model parameters for *m* = 2 layers treating the sample as a step change between the bulk properties of the polymers. The frequency dependence of the modulus *G̃* is parametrized according to eq 17, where *G*_f_^′^ and *G*_f_^″^ for the PB layer at the fundamental *n* = 1 harmonic are fit, along with the PB layer thickness *h*_PB_, holding the exponents β′ and β″ fixed. (The PS layer parameters, *G̃*_PS_ and *h*_PS_, were first individually fit to a measurement of the single layer PS film prior to adding the PB layer.) The density ρ was also held fixed at the individual layers’ respective bulk values. (b) and (c) Graphs of the Δ*f*(*n*) and ΔΓ(*n*) data for six nominally identical samples (gray open diamonds), along with their average and standard deviation (solid black diamonds). The results of the model best fit to these averaged values are highlighted as cyan curves giving *G*_f_^′^ = 2 MPa and *G*_f_^″^ = 3.3 MPa for the PB layers, where *G*_f_^′^ = 1.9 GPa and *G*_f_^″^ = 70 MPa were obtained for the individually fit PS layers.

Figure 5 demonstrates these fits of the QCM transfer-matrix model with *m* = 2 layers to the PS/PB bilayer film at *t* = 0. The data for the individual resonance and dissipation shifts, Δ*f*(*n*) and ΔΓ(*n*), across the six nominally identical samples are shown along with their average and standard deviation. It is these averages across the different harmonics *n* that are simultaneously fit together to the QCM transfer-matrix model. Analyzing the data in this manner allows the fitting to properly weight the relative sample-to-sample variability of each harmonic’s Δ*f* and ΔΓ using the standard deviation calculated from the results of all the samples. For this *t* = 0 initial state, the interfacial width is narrow at ≈1 nm,^54,74^ and the *T*_g_(*z*) and *G̃*(*z*) profiles are expected to be effectively sharp changes between the two polymer layers with bulk properties.^11,34^ As such, we model the system as a two-layer step change between the bulk properties of the polymers. From this fit to the *m* = 2 layer model, what we obtain is the PB layer’s *n* = 1 storage *G*_f_^′^ and loss *G*_f_^″^ modulus from eq 17, along with the PB layer thickness *h*_PB_. The *G̃*_PS_ and *h*_PS_ are held fixed at the values that were first fit to the single PS underlayer. The density ρ and exponents β′ and β″ for the two layers are also held fixed at their respective bulk values.

The experimental resonance and dissipation shifts at this initial *t* = 0 state in Figure 5 define the Δ*f*_*t*=0_(*n*) and ΔΓ_*t*=0_(*n*) quantities that we will use as the reference for the subsequent evolution in Δ*f*(*n*) and ΔΓ(*n*) with annealing at 120 °C.

#### 3.2.2 Evolution of Resonance due to Annealing at 120 °C

**Figure 6:**
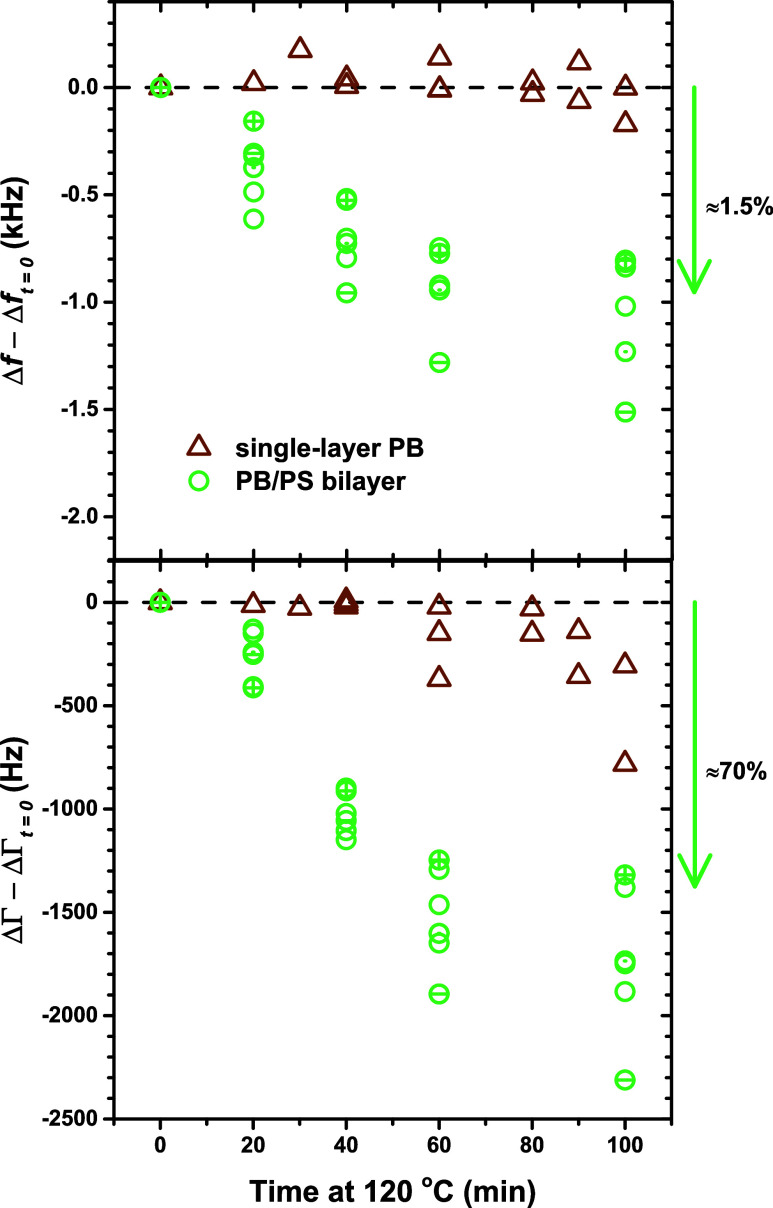
Evolution of the Δ*f* and ΔΓ resonance shifts for the *n* = 7 harmonic with annealing time at 120 °C plotted relative to the initial values at *t* = 0. PS/PB bilayer data (open green circles) are compared to data collected on single-layer PB samples (open brown triangles); data for 6–7 nominally identical samples are shown. An approximate change was observed in the bilayers of ≈70% in ΔΓ, relative to ≈1.5% in Δ*f*. Different interior symbols in the green circles highlight individual samples which exhibit high, low, and intermediate changes on annealing.

In Figure 6, we graph the evolution of Δ*f* and ΔΓ for the *n* = 7 harmonic with annealing at 120 °C, relative to the resonance and dissipation measured for the given sample at the initial *t* = 0 state, Δ*f*_*t*=0_ and ΔΓ_*t*=0_. This focus on Δ*f* – Δ*f*_*t*=0_ and ΔΓ – ΔΓ_*t*=0_ for each individual sample allows us to filter out much of the sample-to-sample variability, primarily associated with the layer thickness, and accentuates the changes that occur in the sample with annealing at 120 °C under vacuum. At the *n* = 7 harmonic, a large decrease in ΔΓ of ≈1500 Hz is observed, corresponding to a ≈70% change relative to the initial ΔΓ_*t*=0_ ≈ 2100 Hz. In contrast, the decrease in Δ*f* with annealing is only ≈1 kHz relative to the initial Δ*f*_*t*=0_ ≈ 65 kHz, representing a tiny ≈1.5% change. Each sample exhibits a consistent trend where the changes in Δ*f* – Δ*f*_*t*=0_ and ΔΓ – ΔΓ_*t*=0_ of the PS/PB samples occur over the first 40–60 min of annealing, and then appear to saturate. We note that the variation present in the Δ*f* – Δ*f*_*t*=0_ and ΔΓ – ΔΓ_*t*=0_ data largely still represents differences across samples, where some samples exhibit larger or smaller changes in Δ*f* and ΔΓ with annealing. This could reflect slight differences in the initial ≈1 nm interfacial width established between the PS/PB layers at room temperature, as we believe the degree of viscoelastic coupling between the layers are strongly dependent on the interfacial width. For example, a smaller initial interfacial width would likely result in a larger change in Δ*f* – Δ*f*_*t*=0_ and ΔΓ – ΔΓ_*t*=0_ with annealing as the PS/PB interface evolves to the ≈5 nm equilibrium value. In addition, a variation in PB layer thickness will impact the magnitude of the initial starting dissipation and therefore the degree to which it can change.

We are interested in understanding how the changes in the resonance and dissipation shifts with annealing at 120 °C reflect the changes in viscoelastic properties of the PS/PB bilayer films. One possible concern is that annealing at 120 °C could theoretically also lead to thermal degradation of the PB layer as this temperature is over 200 °C higher than the bulk *T*_g_ of our PB. In contrast, the PS layer is quite stable at 120 °C. To assess this possibility, we include control measurements on single-layer PB films in Figure 6, where the change in Δ*f* and ΔΓ with annealing at 120 °C under vacuum for the *n* = 7 harmonic is similarly plotted relative to initial measurements at Δ*f*_*t*=0_ and ΔΓ_*t*=0_ for the PB films. We find the Δ*f* and ΔΓ data are stable with annealing at 120 °C up to approximately 60+ min where a small decrease in ΔΓ is observed, along with increased variability in the data. It is possible this change in the PB layer may have also contributed to the increased variability observed in the PS/PB bilayer data and some of the continued reduction at 60+ min. Experiments on PB degradation by Chiantore et al. showed that after an hour of annealing at 192 °C under vacuum little to no *cis*-1,4 bond loss was found, but ∼5% *trans*-1,4 bond degradation did occur.^80^ Chain fragmentation was only observed at temperatures higher than 330 °C. From this, we conclude that thermal degradation of the PB should be minimal, especially for times up to 40–60 min.

The specific annealing time scale needed for the PS/PB interface to reach its equilibrium at 120 °C is unknown, but the PS/PB interfacial composition data by Genzer and Composto compared annealing at low and high temperatures.^54^ They used both neutron reflectivity and low-energy forward recoil spectrometry (LE-FRES) to study the compositional profile of PS/PB bilayer samples composed of ≈60 nm thick layers of dPS or PS/dPS mixture floated atop ≈400 nm thick PB layers. Their molecular weights and PB microstructure were comparable to our present study. Prior to any high temperature annealing, after their samples were held overnight at 50 °C under vacuum, neutron reflectivity indicated an initial interfacial roughness of 1.5 nm. This suggests our initial PS/PB bilayers, held at only 25 °C for 20 h, would not be expected to have a PS/PB interfacial width more than ≈1 nm.^54,55^ The PS/PB bilayer samples by Genzer and Composto were then measured after several days of annealing at 175 °C. Neutron reflectivity indicated an interfacial width of 3.6 nm with a volume fraction profile having a PS/PB interface spanning ≈5 nm.^54^ The LE-FRES reported a comparable value of 6.0 ± 3.5 nm. Genzer and Composto found these values to be in good agreement with expectations from self-consistent field theory calculations using literature values for the PS/PB χ interaction parameter.^54,57^ For our annealing temperature at 120 °C, the weak temperature dependence of χ might suggest a slightly narrower interfacial width by ≈0.2 nm.^34^ Thus, to a reasonable approximation, we conclude that the equilibrium interfacial width of our PS/PB samples is ≈5 nm after annealing at 120 °C.

The progressive time scale for polymer–polymer interface formation is best understood from neutron reflectivity data of PS/PS interfaces,^81−83^ where 40–60 min at 120 °C would be sufficient to reach an interfacial width of ≈5 nm. Data comparing interfacial widths between PS/PnBMA (PnBMA *T*_g_ = 20 °C) and PS/PS that were annealed for 3 h at varying temperatures below and above the *T*_g_ of PS find that polymer–polymer interface formation is primarily limited by the higher *T*_g_ polymer.^55^ This would be consistent with the local *T*_g_(*z* = 50 nm) data in PS next to PSF collected after a series of different annealing time points at 210 °C (25 K above the PSF *T*_g_ = 185 °C).^11^ As shown in Figure 1b, 40 min of annealing established most of the *T*_g_(*z*) perturbation and equilibrium was reached after 60 min.^9,11^ Thus, we would conclude that annealing the PS/PB interface for 40 min at 120 °C likely establishes most of the *T*_g_(*z*) and *G̃*(*z*) perturbation, while true equilibrium may need 60 min. The data shown in Figure 6 would support this conclusion where meaningful changes in Δ*f* – Δ*f*_*t*=0_ and ΔΓ – ΔΓ_*t*=0_ have plateaued by 60 min. More research on the time-dependent development of interfaces between immiscible polymer pairs would be beneficial. In the following analysis, we focus our attention on the 40 min data, where we feel there are no concerns of PB degradation impacting the measured Δ*f* and ΔΓ data.

### 3.3 Analysis of the Annealed Bilayer Data

**Figure 7:**
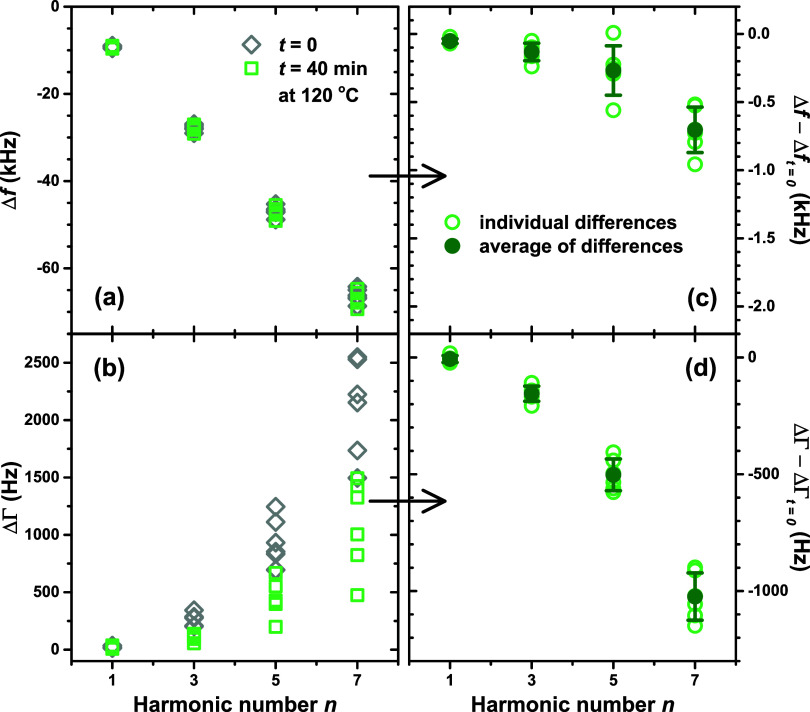
(a) Δ*f*(*n*) and (b) ΔΓ(*n*) collected from PS/PB bilayer samples at *t* = 0 (gray diamonds) and *t* = 40 min (light green squares) of annealing at 120 °C. The change that each individual sample undergoes after *t* = 40 min of annealing relative to *t* = 0 is defined as (c) Δ*f* – Δ*f*_*t*=0_ and (d) ΔΓ – ΔΓ_*t*=0_, plotted as light green open circles, where the dark green solid data and their error bars are the average and standard deviation used for the QCM model fitting.

Focusing on the data at 40 min of annealing at 120 °C, Figure 7 shows the harmonic number *n* (frequency) dependence of Δ*f* and ΔΓ. Following Figure 6, the Δ*f* – Δ*f*_*t*=0_ and ΔΓ – ΔΓ_*t*=0_ are plotted for each of the six individual samples. Reformatting the QCM data in this manner isolates the change in Δ*f* and ΔΓ with annealing and helps account for sample-to-sample variability. It also significantly reduces the impact of the specific layer thicknesses and bulk *G̃* values found for the *t* = 0 case modeled with *m* = 2 layers (Figure 5). The average and standard deviation of these changes in (Δ*f* – Δ*f*_*t*=0_)_exp_ and (ΔΓ – ΔΓ_*t*=0_)_exp_ at each *n* are taken as the data set to be fit by the QCM transfer-matrix model. The main change in the data with annealing that the model will need to capture is the very strong reduction in dissipation (≈50% at *n* = 7 for 40 min) that is consistent across all samples with low variability. The data also show a small decrease in resonance frequency with annealing (only ≈1% at *n* = 7 for 40 min) that is consistent across the different samples.

These experimental quantities of (Δ*f* – Δ*f*_*t*=0_)_exp_ and (ΔΓ – ΔΓ_*t*=0_)_exp_ need model equivalents so that proper comparison can be made for fitting. We define model versions (Δ*f* – Δ*f*_*t*=0_)_model_ and (ΔΓ – ΔΓ_*t*=0_)_model_ that represent perturbations to the modeled step change result that best fit the *t* = 0 initial state data (Figure 5). The model treats the change in Δ*f* and ΔΓ from this initial *t* = 0 state as arising from a gradient in modulus. To write eq 3 in terms of Δ*f* – Δ*f*_*t*=0_ and ΔΓ – ΔΓ_*t*=0_, we add and subtract (Δ*f*_*t*=0_ + *i*ΔΓ_*t*=0_)_model_:
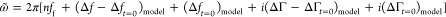
19Formally in the code, Δ*f* and ΔΓ are treated as a single complex quantity: Δ*f̃* = Δ*f* + *i*ΔΓ. Equation 19 is the same as eq 3, only now the variable that is solved for and compared to experimental data is (Δ*f̃* – Δ*f̃*_*t*=0_)_model_ instead of Δ*f̃*_model_. (Δ*f*_*t*=0_)_model_ and (ΔΓ_*t*=0_)_model_ are held constant at the model result values best fit to the average *t* = 0 data shown in Figure 5. With this adjustment in parametrization of ω̃, we can now focus on identifying what changes in *G̃*(*z*) from the original step change model at *t* = 0 are required to produce the experimentally observed changes in Δ*f* and ΔΓ upon annealing.

Fitting is done by minimizing a χ^2^ error function of the form χ_tot_^2^ = χ_*f*_^2^ + χ_Γ_^2^:
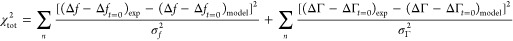
20σ_*f*_ and σ_Γ_ are the standard deviations of the experimental data sets for (Δ*f* – Δ*f*_*t*=0_)_exp_ and (ΔΓ – ΔΓ_*t*=0_)_exp_, respectively.

Use of the transfer-matrix model starts by choosing a number of layers *m* to divide up the PS/PB bilayer system. A modulus gradient can then be assigned between the bulk *G̃*_PS_ and *G̃*_PB_ values determined from the *t* = 0 fit. This gradient is parametrized by the hyperbolic tangent given in eq 18. *G̃* values at each layer are simply assigned according to this functional form. Meanwhile the local density ρ(*z*) is treated as a step-change. As a result, we are left with only two fit parameters: the width *w* and asymmetry μ of the modulus gradient.

The matrix product series in eq 11 has its length set by *m*, and it is used to define the matrix elements *a*, *b*, *c*, and *d* in eq 16. Material properties are incorporated into the matrix elements of each layer through  and  as shown in eq 9. Equation 16, which represents the entire model of the bilayer, is solved by Newton root-finding to identify the corresponding (Δ*f* – Δ*f*_*t*=0_)_model_ and (ΔΓ – ΔΓ_*t*=0_)_model_. The χ_tot_^2^ error is then determined from eq 20. The parameters *w* and μ are iteratively varied so that their values which give the least error can be identified.

A large amount of matrix inversion and matrix multiplication is entailed in this fitting protocol, particularly when the parameter values are iteratively varied due to how many times the root-finding algorithm must be run on eq 16. To keep the computational runtime to a reasonable time-scale that does not exceed some small number of days for each fit, or especially for the generation of error landscapes, we make two optimizations to the assignment of model layers. First, we focus resolution of the *G̃*(*z*) gradient near the PS/PB interface. As the gradient is unlikely to extend more than a few hundred nanometers into the PS, the >1000 nm PS layer is expected to include a large region exhibiting bulk-like behavior, thus it is beneficial to avoid having resolution of the *G̃*(*z*) gradient wasted on that region. Second, we limit the number of model layers to a quantity that we have optimized at *m* = 9.

**Figure 8:**
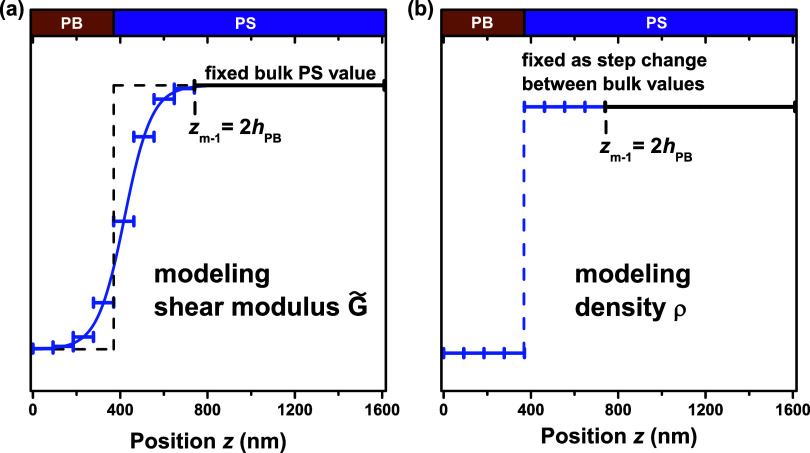
(a) A representation of an arbitrary *G̃*(*z*) gradient model under the refined layer model construction that optimizes resolution about the PS/PB interface. The cutoff *z*_*m*–1_ marking the top of a large layer treated as bulk PS, is chosen to be at 2*h*_PB_ = 760 nm. The 760 nm region of focus around the PS/PB interface always has an equal number of layers on each side by imposing that *m* is odd. (b) The symmetric layer distribution about the interface enables a step change to be easily set for the density.

Figure 8 shows the update to the layer model chosen to optimize the resolution of *G̃*(*z*) about the polymer–polymer interface, as well as create a simple way to keep ρ(*z*) as a step change. We chose to set the cutoff *z*_*m*–1_ = 2*h*_PB_, and keep *m* odd. From the *h*_PB_ fit at the *t* = 0 case of 380 nm, *z*_*m*–1_ is set to 760 nm. This position corresponds to the top of the wide layer that is assigned the bulk PS value for *G̃*. An odd-valued *m* with this choice of *z*_*m*–1_ ensures that there are always an equal number of modeled layers on either side of the interface across the focused 760 nm region. With it now imposed that some *z*_*j*_ always resides at the interface, ρ(*z*) can be easily assigned a step change at the interface. This also allows the *G̃*(*z*) profile to be modeled accurately as sharp or broad in an unbiased manner, where *w* and μ can take on any value. To verify the validity of these optimizations, we varied the cutoff *z*_*m*–1_ from 760 to 1330 nm, keeping the model layer thicknesses fixed by adding additional layers. By running this series of long individual test cases to get χ_tot_^2^ error, we found that χ_tot_^2^ does not change by more than ≈1%, even in the extreme case of a very wide and asymmetric gradient (*w* = 500 nm, μ = 400 nm). The number of model layers *m* was also increased while keeping the cutoff *z*_*m*–1_ fixed at 2*h*_PB_, finding great consistency for *m* = 9 and beyond in the shape of the χ_tot_^2^ landscape associated with comparison to the experimental data collected on annealed samples, as well as in the associated best fits. Best fit values for *w* and μ did not change by more than a nanometer comparing *m* = 9 to *m* = 11 layers. The saturating effect of adding more layers implies that this approach is an effective proxy for modeling a continuous profile.

**Figure 9:**
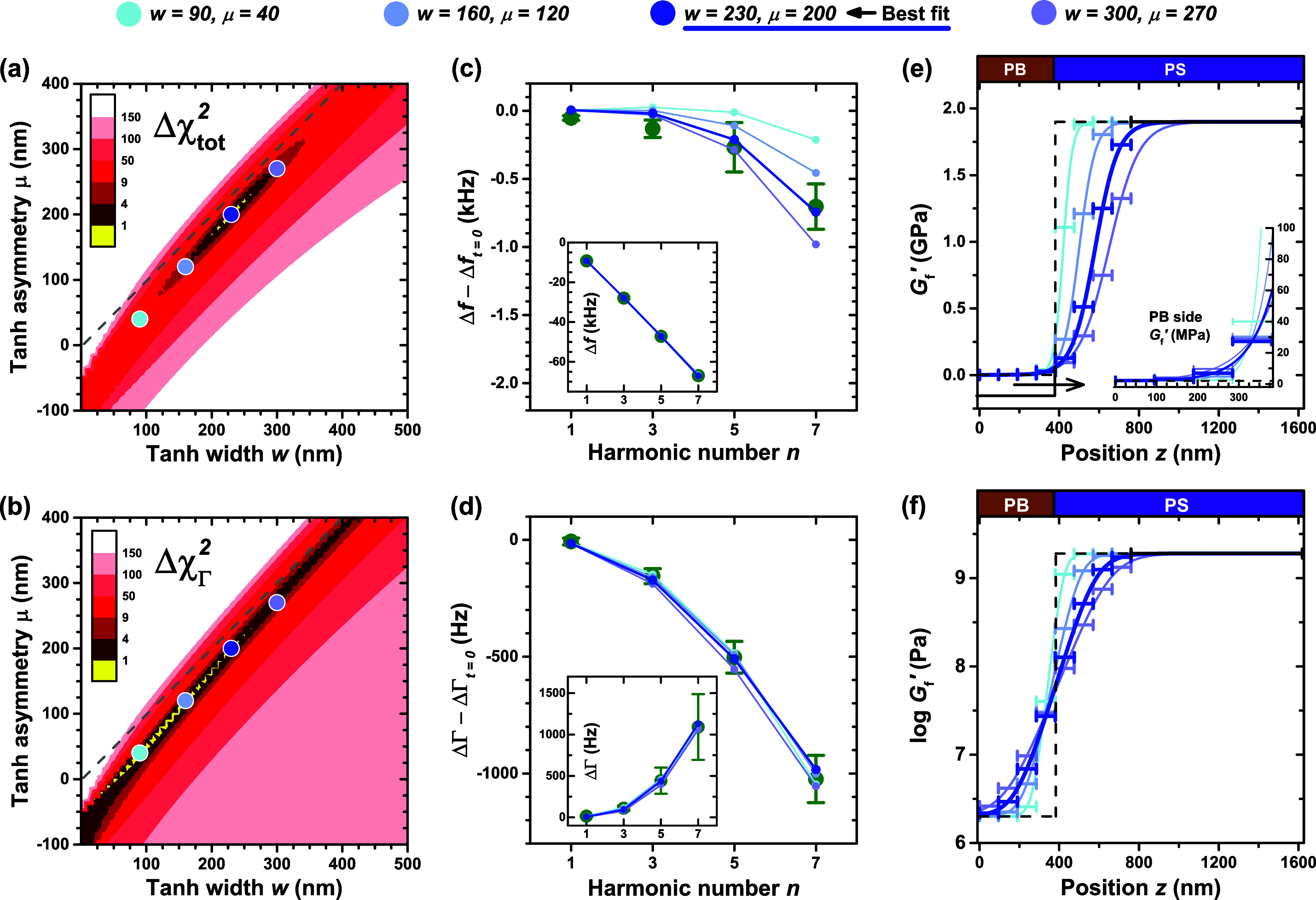
Results from fitting the QCM transfer-matrix model to the experimental Δ*f* – Δ*f*_*t*=0_ and ΔΓ – ΔΓ_*t*=0_ obtained after *t* = 40 min of annealing the PS/PB bilayer at 120 °C. Heat maps showing the difference from the minimum in (a) χ_tot_^2^ (eq 20) and (b) only the χ_Γ_^2^ component, resulting from different width *w* and asymmetry μ values for the hyperbolic tangent model of *G̃*(*z*) (evaluated using *m* = 9). Gray dashed line corresponds to the 1:1 line μ = *w*, demonstrating the minimum lies where *w* > μ and μ is positive such that the *G̃*(*z*) asymmetry is toward the PS side in agreement with the *T*_g_(*z*) profile. Four locations of model curve parameter values spanning the χ^2^ valley of best fit have been assessed in detail, with the best fit *G̃*(*z*) model being *w* = 230 nm, μ = 200 nm (solid blue dot, thick blue curve). Experimental data for (c) Δ*f* – Δ*f*_*t*=0_ and (d) ΔΓ – ΔΓ_*t*=0_ at each measured harmonic are shown against the predictions from the assortment of *G̃*(*z*) models. (e) The *G*_f_^′^(z) component of *G̃*(*z*) is plotted to visualize the hyperbolic tangents describing the modulus profile across the PS/PB interface. The range of models spanning the valley of best fit are shown to identify what consistency and variability exist between them. Bulk PS and PB shear modulus values are not recovered until ≈100+ nm from the interface on either side. The inset highlights the consistency at the interface and on the PB side. The value of *G*_f_^′^ at the interface is only ≈50–100 MPa, considerably below the average of the PS and PB *G*_f_^′^ values. (f) When plotted on a logarithmic scale, the *G̃*(*z*) curves are notably symmetric in log *G*_f_^′^ with *G*_f_^′^ at the interface close to the geometric mean that would optimize impedance matching.

The core analysis performed in this study is described in detail in Figure 9 for the data collected after 40 min of annealing at 120 °C. As the fitting of this QCM data to a modulus gradient is fairly complex, we start by generating heat maps of the error landscape to get a sense of the terrain around the valley of best fit. The error landscape heat maps display the metric Δ*χ*^2^ = χ^2^ – χ_min_^2^, where Figure 9a uses the total χ^2^ given in eq 20. In Figure 9b we choose to focus on only the χ_Γ_^2^ component, as the majority of the change in the data on annealing at 120 °C occurred in ΔΓ. Both heat maps have been color coded such that yellow corresponds to one standard deviation (68.3% confidence interval in each parameter), the darkest red region represents the 95% confidence interval, the next darkest is 99.7%, and further contours were simply chosen to display the error landscape more broadly. These heat maps which vary the modulus gradient asymmetry parameter μ versus the width parameter *w* show that the valley of best fit is sloped such that as *w* increases, asymmetry in the gradient is clearly demanded with the value of μ also increasing. These heat maps suggest that a symmetric gradient about the composition profile would be inaccurate to describe the modulus profile. In addition, the direction of asymmetry in the positive μ direction is toward the glassy PS domain, in agreement with the *T*_g_(*z*) profile. Although the asymmetry of the gradient μ appears nearly as large as the width *w*, the valley of best fit is consistently below the 1:1 line μ = *w*, indicating that the *G̃*(*z*) profile is slightly wider than asymmetric.

The landscape of Δ*χ*_tot_^2^ shows a well-defined minimum at the best fit of *w* = 229.56 nm and μ = 200.12 nm (displayed in solid blue) obtained using *m* = 9 layers. For verification, we also generated coarse grained heat maps for the *m* = 11 case and observed the same overall shape in the error landscapes with the best fit values remaining unchanged at *w* = 229.64 nm, μ = 200.88 nm. This informs us that *m* = 9 analysis is sufficient to emulate a smooth *G̃*(*z*) gradient, and justifies our choice to use that number of layers to enable efficient generation of the high resolution heat maps in Figure 9. In Figure 9c and d, we can see that the predictions associated with the best fit agree well with the experimental data, displayed in terms of Δ*f* – Δ*f*_*t*=0_ and ΔΓ – ΔΓ_*t*=0_, with the insets showing the absolute Δ*f* and ΔΓ. We note that the *G̃*(*z*) gradient models do not fully capture the Δ*f* – Δ*f*_*t*=0_ data shown in Figure 9c at the *n* = 1 and 3 harmonics, even though it is within the symbol of the Δ*f*(*n*) data shown in the inset. We have investigated possible causes for this tiny consistent shift in Δ*f* between *t* = 0 and *t* = 40 min of annealing, but have been unable to identify any systematic error as its origin, ruling out change in mass, slip, a long-range density gradient, or PB oxidation/degradation.

It is informative to examine other positions along the valley of best fit in the error landscapes. These positions are highlighted in light blue in Figure 9a and b, and are similarly associated with the light blue prediction curves in Figure 9c and d. We find that the wide valley of best fit associated with χ_Γ_^2^ in Figure 9b identifies a range of models that all fit the experimental dissipation ΔΓ – ΔΓ_*t*=0_ results well in Figure 9d. However, in Figure 9c it is clear the solid blue curve captures the experimental Δ*f* – Δ*f*_*t*=0_ result significantly better, hence the more precise minimum in the χ_tot_^2^ landscape.

Figure 9e plots the different *G̃*(*z*) curves representing the range of models across the valley of best fit, in which commonalities are observed that allowed these curves to all fit the data reasonably well. Notably the different tanh curves in Figure 9e appear nearly identical on the PB side, having just the right *w* and μ values to behave very similarly. The inset of Figure 9e highlights how well these distinct curves all line up on the PB side. For example, the value of *G*_f_^′^ for the ≈100 nm layer adjacent to the interface on the PB side is reliably ≈30 MPa, and at the PS/PB interface, *G*_f_^′^ is ≈50–100 MPa, with the exception of the curve furthest from the χ_tot_^2^ minimum. In contrast, the wide range of behavior on the PS side indicates that there is considerably less sensitivity to modulus variation in this regime. We believe this is due to the fact that the thin model layers (≈100 nm in thickness for *m* = 9) are not very sensitive to variation in *G*_f_^′^ when it is in the GPa regime. The lack of sensitivity in this regime where *G*′ ≫ *G*″ is why thick PS layers are experimentally needed to measure modulus with QCM at MHz frequencies.^48^ In contrast, much thinner films can be used to measure the modulus of PB, having a *G*_f_^′^ value in the MPa regime where *G*′ ≈ *G*″.

Interestingly, the *G*_f_^′^ value of ≈50–100 MPa at the PS/PB interface is significantly below the average of the two bulk *G*_f_^′^ values at ≈1 GPa. This indicates that the simplest assumption of an average intermediate modulus made by Gagnon et al.^34^ when fitting a 3-layer model to these types of data was not accurate. On the linear modulus scale shown in Figure 9e, the low value of the modulus at the PS/PB interface speaks to the strong asymmetry of the *G̃*(*z*) gradient, where the best fit value of *w* = 230 nm and μ = 200 nm demonstrates that the *G̃*(*z*) curve is nearly as asymmetric as it is wide. The *T*_g_(*z*) gradient repeatedly observed in previous experiments did not have asymmetry to this extent, with *T*_g_(*z*) gradient parameters being *w* = 209 nm and μ = 75 nm for PS/PB.^10−12^ The fact that both the *G̃*(*z*) and *T*_g_(*z*) gradients are asymmetric toward the glassy PS side with comparable widths suggests these material properties are locally linked in some fashion, but the strong difference in asymmetry indicates that local correlation may not be as simple as one might naively expect. Similar to *T*_g_(*z*), the bulk *G*_f_^′^ value of PB appears to only be recovered at ≈100+ nm from the PS/PB interface. On the PS side, despite the high variability, a minimum of ≈200+ nm to recover the PS bulk *G*_f_^′^ is evident as well.

Considering the drastic asymmetry and strong influence of the seemingly minuscule change on the PB side, in Figure 9f we examine the *G̃*(*z*) curves on a logarithmic modulus scale. Surprisingly, the log *G̃*(*z*) curves are found to be symmetric about the PS/PB interface. In fact, the best fit log *G̃*(*z*) curve can be well described by a hyperbolic tangent that is 371 nm wide and offset from the interface by only 3 nm. This observation suggests that the naive assumption that one would naturally make that the interface should have the average modulus value on a linear scale is not correct, and that looking at modulus on a linear scale may be misleading.

**Figure 10:**
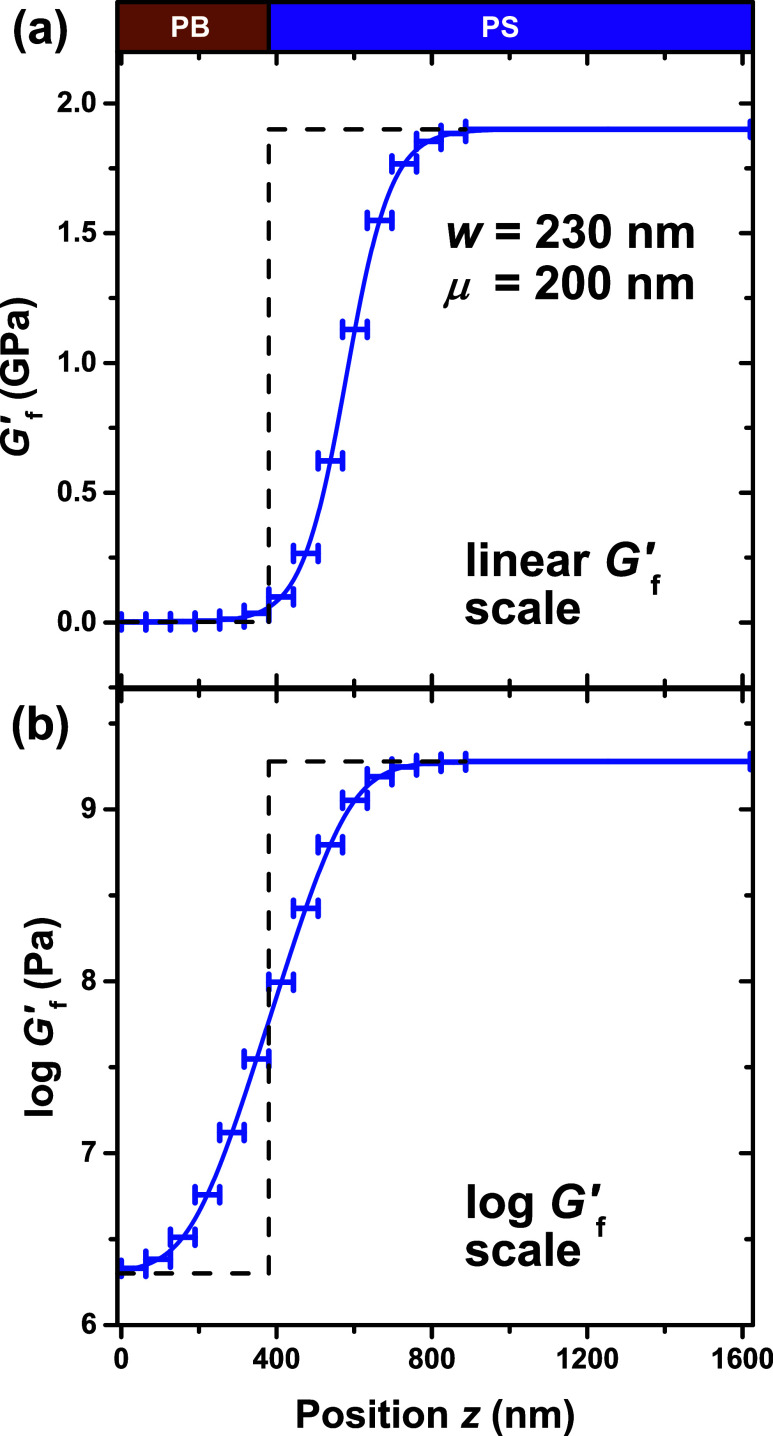
Hyperbolic tangent *G̃*(*z*) model with width *w* = 230 nm and asymmetry μ = 200 nm that was best fit to the data collected at *t* = 40 min of annealing at 120 °C, plotted on either linear (a) or logarithmic (b) modulus scales, where the transfer matrix model has been evaluated using *m* = 15 layers and *z*_*m*–1_ is extended farther into the PS domain.

In Figure 10 we highlight the *G̃*(*z*) layer model corresponding to the best fit *w* = 230 nm and μ = 200 nm curve to the experimental *t* = 40 min data from Figure 9. However, in Figure 10, the transfer matrix model is evaluated at a higher resolution with *m* = 15 layers, where the *z*_*m*–1_ cutoff is extended to *z* = 887 nm in order to ensure the full gradient is sliced into layers. This simultaneously enhances the resolution on both sides of the PS/PB interface. The χ_tot_^2^ value for this high resolution model is only different by 0.2 (≈1%) compared to the *m* = 9, *z*_*m*–1_ = 760 nm layer model from Figure 9 that uses the same *w* and μ values.

The physical importance to the finding that log *G̃*(*z*) is symmetric may relate to the fact that the transmission of acoustic shear waves between domains is optimized when an impedance matching layer has an impedance *Z̃* = (*ρG̃*)^1/2^ corresponding to the geometric mean *Z*_geo_ = (*Z*_1_*Z*_2_)^1/2^ of the neighboring domains.^84^ This means that in between two domains one would want an intermediate layer with modulus *G*_geo_ = (*G*_1_*G*_2_)^1/2^ that would optimize the impedance:
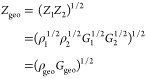
21The average of the bulk log *G*_f_^′^ values is the geometric mean *G*_f_^′^ = 62 MPa, different by only 3 MPa from the best fit modulus *G*_f_^′^ value at the interface of 59 MPa. This observation would support the hypothesis by Gagnon et al.^34^ that long-ranged coupling of local properties occurs via impedance matching of acoustic shear waves.

How might this *G̃*(*z*) modulus profile measured by QCM relate to the *T*_g_(*z*) profile previously measured by pyrene fluorescence for the PS/PB bilayer system? The QCM measurements collected at room temperature reflect the value of the modulus at MHz frequencies (5–35 MHz). We have previously shown that this frequency dependent modulus obtained by QCM is consistent with time–temperature shifted rheometry data for the same polymer from the literature collected at low frequencies (<10^2^ s^–1^).^48^ In contrast, *T*_g_ corresponds to the temperature on cooling at which the system falls out of equilibrium. This cooling rate dependent *T*_g_ value reflects the time scale when the occurrence of α-relaxations becomes slower than the time scale needed to equilibrate the system before further cooling occurs. For the experimental cooling rate of 1 K/min that the pyrene fluorescence measurements were done at, this typically corresponds to time scales of τ_α_(*T*) ∼ 100 s. α-relaxations associated with *T*_g_ correspond to an instantaneous hopping event involving the cooperative rearrangement of multiple polymer segments. It is still an open question what local property of the glass dictates the probability of an α-relaxation event occurring at a given location. However, several theoretical formulations suggest that a dominant element is the high frequency modulus corresponding to the vibrational potential at GHz-THz frequencies associated with the boson peak.^49,50,85−90^ Recent computational work has shown that soft regions of the vibrational potential result in collective quasi-localized oscillations that appear to be associated with the locations where α-relaxation events end up occurring.^51,88,89,91,92^ Such quasi-localized excitations (QLEs) in soft potential regions would be expected to interact with propagating acoustic waves of equivalent energy,^51,88,93,94^ which correspond to acoustic waves in the vibrational spectrum near the boson peak with wavelengths λ ∼ 5 nm.^34,95^

The similarity in breadth and asymmetry between the QCM measured *G̃*(*z*) gradient and the *T*_g_(*z*) gradient suggest that the material properties of modulus and *T*_g_ remain correlated locally as in bulk systems. This correlation and the time–temperature superposition of modulus master curves likely signify that the high frequency modulus correlated with α-relaxations also exhibits a similar gradient. Our finding that the *G̃*(*z*) modulus profile is symmetric on a log scale where impedance matching conditions are maximized at the geometric mean, corresponding to the average in log *G̃*, may inform us about the underlying mechanism of this long-range gradient. The physics of acoustic wave transmission with λ ∼ 5 nm across a broadened ≈5 nm glassy–rubbery polymer interface would be expected to be symmetric, where impedance matching conditions would dictate which acoustic waves that are part of the vibrational density of states are the ones most likely to propagate across the interface. This transmission of high frequency acoustic waves could couple the vibrational spectra of the two polymer domains resulting in a gradient in vibrational potential, i.e., high frequency modulus of the material. Wave penetration typically decays exponentially with depth, consistent with the hyperbolic tangent profile we fit the data to. The mean free path of acoustic waves, i.e., the distance over which the wave travels before some scattering event occurs, has been measured to be hundreds of nanometers in glasses.^96^ Thus, it seems reasonable that high frequency acoustic wave transmission can provide a long-range mechanism by which acoustic waves could trigger density fluctuations in neighboring polymer domains resulting in α-relaxations and associated *T*_g_(*z*) and *G̃*(*z*) gradients. The causality of this plausible phenomenon would be that impedance matching conditions for high frequency acoustic waves created by the formation of the broadened ≈5 nm polymer–polymer interface couples the vibrational density of states near the boson peak, where acoustic wavelengths λ ∼ 5 nm create a gradient in vibrational potential and high frequency modulus that result in the *T*_g_(*z*) gradient and the measured *G̃*(*z*) gradient at lower frequencies.

## 4 Conclusions

In the present work, we have built upon our physically intuitive continuum mechanics model^34,48^ for fitting QCM Δ*f*(*n*) and ΔΓ(*n*) data by using a transfer-matrix analysis that matches acoustic boundary conditions across an arbitrary number of discrete modeled layers. This new approach to modeling QCM data enables the determination of high resolution depth-dependent profiles in local modulus *G̃*(*z*) for glassy–rubbery bilayer films. By studying QCM resonances from PS/PB bilayer films upon annealing the glassy–rubbery interface toward equilibrium, we have observed a consistent large decrease in dissipation ΔΓ with annealing time indicative of large changes in the viscoelastic properties of these bilayer films. Using our new QCM analysis method, we have mapped the evolution of the local modulus *G̃*(*z*) profile upon annealing of the interface, providing new insight into local property changes and the previously observed local *T*_g_(*z*) profiles across such systems.

Fitting a hyperbolic tangent curve to the gradient in *G̃*(*z*) that develops after 40 min of annealing at 120 °C, the best fit yields a width *w* of 230 nm and an asymmetry μ of 200 nm toward the PS side. The asymmetry direction and breadth of this gradient in shear modulus across the PS/PB interface generally corroborates what has been previously observed for *T*_g_(*z*). This suggests that *T*_g_ and modulus remain correlated locally across the glassy–rubbery interface, and not only in bulk. The asymmetry is notably stronger in *G̃*(*z*), where a characterization of the shape of the error landscape corresponding to the QCM model clearly indicates a strong asymmetry toward the PS side is required in order to explain the data. The necessity of this modulus gradient asymmetry was obscured in previous work by limitations in what information could be extracted by the simpler modeling techniques employed.^34^ The strong asymmetry of the *G̃*(*z*) gradient identified by the new transfer-matrix model corresponds to a best fit (5 MHz) elastic shear modulus at the PS/PB interface equal to 59 MPa, far below the arithmetic mean of the two bulk shear modulus values.

A key observation we find is that the *G̃*(*z*) gradient is symmetric on a logarithmic modulus scale, where the G′ value at the PS/PB interface is extremely close to the geometric mean *G*_geo_ = (*G*_PS_^′^*G*_PB_^′^)^1/2^ of the bulk values. This symmetry seen on a logarithmic scale may be indicative of the physical mechanism responsible for the broad property gradients. Transmission of acoustic waves between two domains is optimized when an intermediate impedance matching layer has an impedance *Z̃* ∝ *G̃*^1/2^ equivalent to the geometric mean.^84^ Perhaps this symmetric logarithmic interaction in modulus across the interface is responsible for indirectly imposing the asymmetric behavior previously observed in *T*_g_(*z*) due to the relationship between *T*_g_ and modulus.

Finally, this new transfer-matrix analysis of our continuum mechanics QCM model could be easily adapted to fit QCM data studying other systems with depth-dependent gradients in viscoelastic properties or density, provided the geometry of the sample has sufficient QCM sensitivity to characterize a distinguishable viscoelastic response. In Supporting Information, we link to a Github repository PyQCM that makes this Python code publicly available.

## Supporting Information Available

Supporting Information PDF contains Github repository PyQCM’s readme file and link to the Python code for the QCM transfer-matrix analysis. Link to Github repository PyQCM: https://github.com/aacoutu/PyQCM (PDF)
