# Supplementary material for: Analyzing QCM Data Using a New Transfer-Matrix Model: Long-Ranged Asymmetric Gradient in Shear Modulus Identified Across Immiscible Glassy–Rubbery Polymer Interface
Source: Macromolecules. 2025 Mar 19;58(7):3520–36. doi: 10.1021/acs.macromol.4c02847 (PMC11984312; doi:10.1021/acs.macromol.4c02847)
Supplement: Supplementary file 1 — ma4c02847_si_001.pdf [file ma4c02847_si_001.pdf]

SUPPORTING INFORMATION

Analyzing QCM Data Using a New Transfer-Matrix Model: Long-Ranged Asymmetric Gradient in Shear Modulus Identified Across Immiscible Glassy-Rubbery Polymer Interface

Alexander A. Couturier, Justin C. Burton, Connie B. Roth*

Department of Physics, Emory University, Atlanta, Georgia, 30322 USA

*To whom correspondence should be addressed. Email: cbroth@emory.edu

Link to Github repository PyQCM: https://github.com/aacoutu/PyQCM

PyQCM Public

Watch 1

main · 1 Branch · 0 Tags · Go to file t · Add file · Code

aacoutu Update README.md — b8ddb39 · 33 minutes ago — 11 Commits

scripts	Add files via upload	4 months ago
README.md	Update README.md	33 minutes ago

README

Included in the "scripts" folder is a set of Python code for running an acoustic transfer-matrix analysis of films measured using a quartz crystal microbalance (QCM). This analysis (in "Modeling_Film_Properties.py") makes use of a continuum mechanics model of shear wave propagation, where the film deposited on the QCM sensor can be modeled using an arbitrary number of layers in order to identify local gradients in viscoelastic properties or density. Our study using this approach can be found at [doi link here]: "Analyzing QCM Data Using a New Transfer-Matrix Model: Long-Ranged Asymmetric Gradient in Shear Modulus Identified Across Immiscible Glassy-Rubbery Polymer Interface," Alexander A. Couturier, Justin C. Burton, Connie B. Roth. In this study, we use the code included here to determine a large gradient in shear modulus across a glassy/rubbery polymer interface of polystyrene (PS) and polybutadiene (PB). Detailed information on the mathematical reasoning underlying the code can be found in our publication.

The input data one needs to provide "Modeling_Film_Properties.py" are the shifts in resonance frequency and dissipation of the QCM sensor as a result of the deposition of the studied film. Such data can be extracted from raw resonance traces using "Circuit_Analysis.py" (also included in the "scripts" folder), where the specific circuit equation used corresponds to our experimental setup. This code analyzes a given set of QCM resonance traces at different harmonics and outputs the associated resonance frequency and dissipation data. For the details of our circuit equation and how it corresponds to our experimental setup, see the publication: "Physically Intuitive Continuum Mechanics Model for QCM: Viscoelasticity of Rubbery Polymers at MHz Frequencies," Yannic J. Gagnon, Justin C. Burton, and Connie B. Roth, Journal of Polymer Science 2022, 60, 244-257 (https://doi.org/10.1002/pol.20210763).
